# Clinician perspectives on successes and challenges in engaging care partners

**DOI:** 10.1002/alz.087977

**Published:** 2025-01-09

**Authors:** Howard Chertkow

**Affiliations:** ^1^ Canadian Consortium on Neurodegeneration in Aging (CCNA), Montreal, QC, Canada; 3560 Bathurst Street Toronto, 3560 Bathurst Street Toronto, ON, Canada; University of Toronto, Toronto, ON Canada

## Abstract

Involvement of caregivers in dementia research is an admirable goal. Involvement of caregivers in care planning for patients and people living with dementia, is essential to good medicine. However, it is important to assess and realistically consider areas where researchers and caregivers, and physicians and caregivers, may disagree. Several kinds of such disagreement and formulating an approach to these will be discussed. Three examples: Involvement of patients in Alzheimer Disease clinical trials. The researcher must be realistic but optimistic in the possible benefits of clinical trials. At the same time, expectations must be managed. It would be naive to consider that caregivers and people with lived experience participate for purely altruistic reasons. Therefore, when a clinical trial fails to produce obvious benefits (the patient may be in a placebo group, or the drug may have limited efficacy), the caregiver often considers withdrawing from the study. How should a researcher approach such a situation? Is there a way for caregivers and patients to be given autonomy, and yet carry clinical trials to the necessary completion? A second example involves prognostication. particularly when (as in Canada) medical assistance in death is now available for dementia patients. Who determines the narrative to be presented to the patient? If the caregiver wishes for greater pessimism or optimism on the part of the physician, is it ethical to diverge from the “scientific” information to accomodate prognostication to the preference of the caregiver? A third complex area is communication. Some caregivers are now requesting that the word “dementia” not be used, because it brings with it stigma. Other families maintain that the new terminology (such as “major neurocognitive disorder”) is mere obfuscation a lacks the clarity needed for communication. Should the language and terminology used be determined by caregivers? Should this be an area of discussion at the beginning of any medical interaction?

We will discuss these and other scenarios where the complexity of the relationship between caregiver and physician/researcher are notable, and suggest approaches and processes to address these.

